# Hepatitis C virus represses the cellular antiviral response by upregulating the expression of signal transducer and activator of transcription 3 through sponging microRNA-122

**DOI:** 10.3892/mmr.2014.2897

**Published:** 2014-11-07

**Authors:** YULIN XIONG, CHANGJIANG ZHANG, JING YUAN, YAN ZHU, ZHAOXIA TAN, XUEMEI KUANG, XIAOHONG WANG

**Affiliations:** Institute of Infectious Diseases of Chinese PLA, Southwest Hospital, Third Military Medical University, Chongqing 400038, P.R. China

**Keywords:** hepatitis C virus, microRNA, signal transducer and activator of transcription 3, interferon, microRNA sponge

## Abstract

microRNAs (miRNAs) are small, non-coding RNAs that inhibit the expression of target protein coding genes at the post-transcriptional level. miR-122 is a liver specific miRNA. Notably, miR-122 is used by the hepatitis C virus (HCV) for triggering viral replication by interacting with the 5′ untranslated region of the HCV RNA. The present study demonstrated that miR-122 inhibited the expression of signal transducer and activator of transcription 3 (STAT3), an antivirus response repressor. The HCV RNA acted as an ‘miRNA sponge’, which upregulated the expression of STAT3 by sealing miR-122. Subsequently, it was confirmed that this miR-122 sponge function of HCV RNA repressed the expression of polyinosinic-polycytidylic acid-stimulated type I interferons. The present study provided a deeper understanding of the complex role of miR-122 in the progression of HCV infection and supported the miR-122 inhibition strategy in anti-HCV infection treatment.

## Introduction

microRNAs (miRNAs) are noncoding RNA molecules, 19–24 nucleotides in length, that repress post-transcriptional gene expression. miRNAs are important in maintaining normal human body physiological conditions and the abnormal expression of miRNA is associated with several human diseases, ranging from psychiatric disorders (1) to various types of malignant cancer (2,3). In addition, they are important in regulating host gene expression in virally infected cells and in types of cancer caused by viral infection (4–7).

miR-122 is a liver specific microRNA and is the most abundantly expressed type of microRNA in the liver (8,9). Previous studies suggest that miR-122 is involved in maintaining the normal function of the liver (10–12). Esau *et al* (13) demonstrated that miR-122 positively regulates lipid metabolism by reducing lipid-associated protein mRNA and that inhibiting the expression of miR-122 attenuates liver steatosis in mice fed a high-fat diet. miR-122 can activate the translation of p53 mRNA through the suppression of cytoplasmic polyadenylation element-binding protein and is involved in cellular senescence (14). miR-122 is also known to be involved in cholesterol synthesis (13,15).

The hepatitis C virus (HCV) is a positive-sense single-stranded RNA virus with a 9.6 kb genome that establishes persistent infections in the liver, eventually leading to cirrhosis and carcinoma. Notably, miR-122 is used by HCV for triggering viral replication by repression of heme oxygenase-1 (16) or by interaction with the 5′ untranslated region (UTR) of HCV RNA (17,18). In addition, miR-122 was found to be dysregulated in different stages of HCV-infected liver tissues and serum (19,20).

The present study identified target genes of miR-122. The effect of HCV mRNA overexpression on expression of target genes was investigated, as well as downstream effects which reduced cellular immune responses to HCV infection.

## Materials and methods

### Reverse transcription quantitative polymerase chain reaction (RT-qPCR)

RT-qPCR analysis was used to determine the relative expression level of miR-122. Total RNA was extracted from the tissues using TRIzol (Invitrogen Life Technologies, Carlsbad, CA, USA) according to the manufacturer’s instructions. The expression level of miR-122 was detected using TaqMan miRNA RT-qPCR. Single-stranded cDNA was synthesized using a TaqMan microRNA Reverse Transcription kit (Applied Biosystems, Foster City, CA, USA) and then amplified using TaqMan Universal PCR Master mix (Applied Biosystems) with an miRNA-specific TaqMan MGB probe, miR-122-5p (Applied Biosystems). U6 snRNA was used for normalization. Each sample was measured in triplicate and the experiment was repeated at least three times for the detection of miR-122.

To detect HCV RNA in plasmid-transfected cells, total RNA was first subjected to reverse transcription using a random hexamer as a primer. The cDNA was then subjected to qPCR and relative mRNA was calculated by normalizing the values of the indicated genes to that of β-actin.

### Cell culture

Huh7 cells (American Type Culture Collection, Manassas, VA, USA) were cultured in Dulbecco’s modified Eagle’s medium (Invitrogen Life Technologies) containing 10% fetal bovine serum (HyClone, Logan, UT, USA), 100 IU/ml penicillin and 10 mg/ml streptomycin (Sigma, St. Louis, MO, USA). All cells were maintained at 37°C in a 5% CO_2_ atmosphere.

### 3′UTR and 5′UTR luciferase reporter assays

To generate the 3′UTR luciferase reporter, a segment of 567 bp 3′UTR from STAT3 was cloned into the downstream of the firefly luciferase gene in a pGL3-control vector (Promega Corp., Madison, WI, USA). The miR-122 mimic and miR-122 inhibitor were synthesized by GenePharma Co., Ltd. (Shanghai, China). A thymidine kinase promoter-Renilla luciferase reporter plasmid (pRL-TK) vector containing Renilla luciferase (Promega Corp.) was co-transfected for data normalization. For the luciferase reporter assays, Huh7 cells were seeded into 48-well plates. Luciferase reporter vectors were co-transfected with the miR-122 mimic or inhibitor using lipofectamine 2000 (Invitrogen Life Technologies). After 2 days, the cells were harvested and assayed using a Dual-Luciferase assay (Promega Corp.). Each treatment was performed in triplicate in three independent experiments. The results were expressed as relative luciferase activity (firefly luciferase/Renilla luciferase).

To generate the 5′UTR luciferase reporter, wild type or mutant HCV RNA 5′UTR was cloned into the upstream of the firefly luciferase gene in pGL3-Basic vector (Promega Corp.). miR-122 mimic and pGL3-Basic-HCV 5′UTR vectors were co-transfected into Huh7 cells and a pRL-TK vector was used for data normalization.

### Western blot analysis

Protein extracts were boiled in sodium dodecyl sulphate/β-mercaptoethanol sample buffer (Sangon Biotech Co., Ltd, Shanghai, China) and 20 μg samples were loaded into each lane of 10% polyacrylamide gels. The proteins were separated by electrophoresis and the proteins in the gels were blotted onto polyvinylidene difluoride membranes (Amersham Pharmacia Biotech, St. Albans, UK) by electrophoretic transfer. Antibodies against the following proteins were used: Monoclonal anti-tubulin (Santa Cruz Biotechnology, Inc., Austin, TX, USA), anti-β-actin (Santa Cruz Biotechnology, Inc.) and anti-STAT3 (Cell Signaling Technology, Inc., Danvers, MA, USA) antibodies (goat anti-human).

### Statistical analysis

Data were analyzed using an SPSS statistical package, version 16 (SPSS, Inc., Chicago, IL, USA) and a two group independent samples t-test. P<0.05 was considered to indicate a statistically significant difference.

## Results

### miR-122 represses the expression of STAT3 by targeting the STAT3 mRNA 3′UTR

miRNA is an important post-transcriptional negative regulator for protein coding genes and may directly target numerous genes. miR-122 is a liver abundant miRNA, which has several functions, including the control of lipid metabolism. To expand on the current knowledge of the function of miR-122 during HCV infection, the present study searched for new target genes using bioinformatics tools. Based on the prediction of the online bioinformatics tool, TargetScan (http://www.targetscan.org/), STAT3 mRNA was identified as a potential direct target of the miR-122 3′UTR.

**Figure 1:**
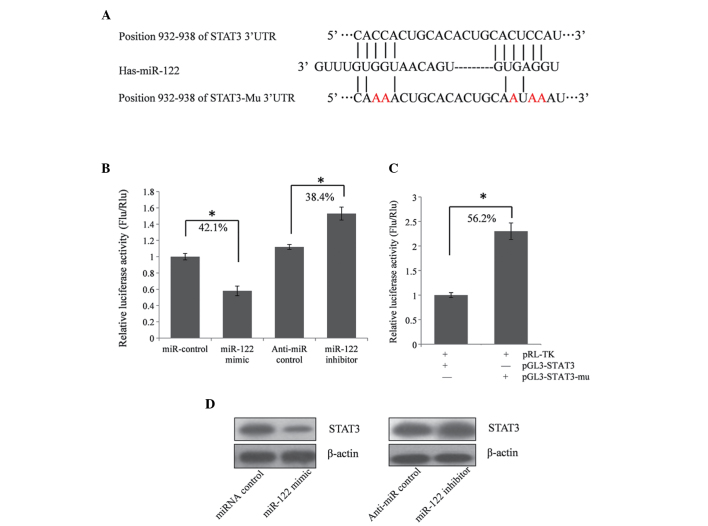
miR-122 suppresses the expression of STAT3 by targeting the STAT3 mRNA 3′UTR. (A) Predicted miR-122 binding site in STAT3 3′UTR. (B) Confirmation of the target gene of miR-122. Huh7 cells were co-transfected with the miRNA control, miR-122 mimic, anti-miR control or miR-122 inhibitor and pGL3-STAT3 for a dual-luciferase assay. PRL-TK containing Rlu was co-transfected for data normalization. (C) Mutation analysis of the miR-122 binding sites. When five nucleotides of the miR-122 binding site were mutated (pGL3-STAT3-Mu), the luciferase activity was significantly increased compared with the wild type STAT3. (D) STAT3 protein level in the miR-122 mimic or inhibitor-treated Huh7 cells was detected by western blot analysis. ^*^P<0.05 vs. controls. miR, microRNA; STAT3, signal transducer and activator of transcription 3; 3′UTR, 3′ untranslated region; PRL-TK, thymidine kinase promoter-Renilla luciferase reporter plasmid; mu, mutant; Rlu, Renilla luciferase; Flu, firefly luciferase.

To validate whether STAT3 is indeed the target gene of miR-122, a 567 bp segment of STAT3 3′UTR containing the predicted miR-122 binding site was cloned downstream in the firefly luciferase reporter gene, in the pGL3 control vector (designated as pGL3-STAT3) for the dual luciferase assay (Fig. 1A). Huh7 cells were co-transfected with pGL3-STAT3 and the miR-122 mimic or inhibitor. Compared with the miRNA control, the luciferase activity was significantly suppressed by miR-122, by ~42.1% (P<0.05; Fig. 1B). Furthermore, the luciferase activity was significantly upregulated by the miR-122 inhibitor compared with the anti-miR control by ~38.4% (P<0.05). These results indicated that miR-122 targeted the 3′UTR of STAT3, leading to a change in firefly luciferase translation.

A seed sequence mutation clone was also used to further confirm the binding site for miR-122 (Fig. 1A). The vector containing a putative miR-122 binding region in the 3′UTR of STAT3 with five mutant nucleotides (designated as pGL3-STAT3-Mu) was used and a wild type STAT3 vector was used as a control. The histogram in Fig. 1C shows that the enzyme activity was reduced by ~56.2% in cells transfected with pGL3-STAT3 compared with pGL3-STAT3-Mu (P<0.05). These data indicated that miR-122 may have suppressed the expression of STAT3 by binding to the seed sequence at the 3′UTR of STAT3 and that STAT3 may be a direct target gene of miR-122.

### miR-122 inhibits endogenous STAT3 expression in Huh7 cells

Although STAT3 was identified as a target gene for miR-122, it remains to be elucidated whether miR-122 is able to regulate endogenous expression of STAT. Huh7 cells were transfected with either miR-122 mimics or an miR-122 inhibitor to establish whether the dysregulation of miR-122 expression affected the endogenous expression of STAT3. Compared with the corresponding control, the protein level of STAT3 was significantly suppressed by the miR-122 mimics and upregulated by the miR-122 inhibitor (Fig. 1D).

### HCV RNA overexpression sponges miR-122 and rescues the expression of STAT3 in Huh7 cells

‘miRNA sponges’ are miRNA competitive inhibitors, which are expressed from strong promoters, containing multiple, tandem binding sites to one or several miRNAs of interest. When vectors encoding these sponges are transiently transfected into cultured cells, sponges can derepress miRNA targets (21).

**Figure 2:**
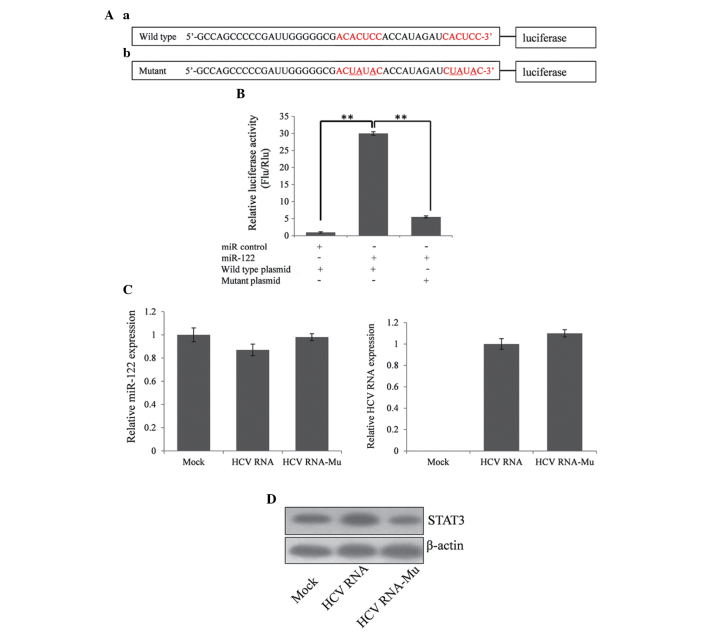
HCV mRNA represses the expression of polyinosinic-polycytidylic acid-stimulated type I interferon by upregulating STAT3. (Aa) Wild type and miR-122 target site mutant HCV mRNA 5′UTR were added prior to the firefly luciferase coding sequence of the pGL3-basic vector. (Ab) A dual luciferase assay was used to detect the interaction between the miR-122 and the HCV 5′UTR. miR-122 enhanced luciferase activity by targeting the HCV 5′UTR. (B) When the binding sites were mutated, the relative luciferase activity was significantly reduced. (C) miR-122 expression reduced when the wild type HCV mRNA was overexpressed, however, the difference was not significant. (D) The expression of STAT3 was upregulated by wild type HCV mRNA rather than the miR-122 target site mutant HCV mRNA, thus, HCV mRNA protects the expression of STAT3 against inhibition from miR-122. ^*^P<0.05, ^**^P<0.01 vs. cells co-transfected with miR112 and wild-type plasmid. HCV, hepatitis C virus; miR, microRNA; 5′UTR, untranslated region; STAT3, signal transducer and activator of transcription 3; Mu, mutant.

To examine whether HCV RNA can protect the expression of miR-122 target genes by interacting with miR-122, the experiment revealing the ability of miR-122 to target HCV mRNA 5′UTR was repeated (Fig. 2A). As shown in Fig. 2B, overexpression of miR-122 enhanced the expression of luciferase compared with the scramble miR-control. When three nucleotides of the miR-122 target sites were changed, the relative firefly luciferase activity was significantly reduced. Transient transfection of the vector encoding 9658 bp HCV genotype 1b RNA into Huh7 cells was performed and RT-qPCR was used to determine the expression of miR-122 24 h after transfection. The present study demonstrated that expression of miR-122 partially reduced compared with the control, however, the difference was not significant (Fig. 2C). Subsequently, the expression of STAT3 was assessed by western blot analysis. Notably, the expression of STAT3 was upregulated by overexpression of HCV RNA compared with the empty vector and the miR-122 target site mutant vector (Fig. 2D), suggesting that HCV RNA protected the expression of STAT3 by absorbing miR-122.

### HCV RNA overexpression inhibits the expression of IFN by rescuing STAT3

**Figure 3:**
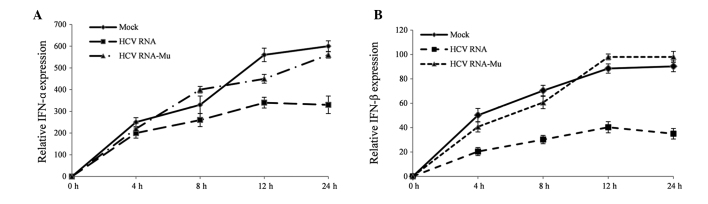
HCV mRNA overexpression represses IFN-α and IFN-β expression. (A) HCV mRNA overexpression repressed the expression of IFN-α in Huh7 cells. (B) HCV mRNA overexpression repressed the expression of IFN-β in Huh7 cells. HCV, hepatitis C virus; IFN, interferon; Mu, mutant.

STAT3 is considered to be a negative regulator of the type I IFN-mediated antiviral response. STAT3 knockdown or knockout cells exhibit enhanced gene expression and antiviral activity in response to IFN-α and IFN-β (22). To examine the response of HCV RNA overexpression on the generation of type I IFN, the present study detected the expression of IFN-α and IFN-β using RT-qPCR at four time points following plasmid and polyinosinic-polycytidylic acid transfection. HCV RNA repressed the expression of IFN-α (Fig. 3A) and IFN-β (Fig. 3B), however, the miR-122 target site mutant HCV RNA did not, suggesting that HCV RNA repressed the cell anti-viral response by absorbing miR-122 directly.

## Discussion

HCV is a positive-sense single-stranded RNA virus. At present, >170 million individuals worldwide are chronically infected with HCV, and cirrhosis and hepatocellular carcinoma induced by HCV infection are life-threatening diseases (23,24). miR-122 is a liver-specific miRNA and its ability to promote, rather than inhibit, HCV RNA function has rendered miR-122 an attractive therapeutic target. In the present study, STAT3 was initially confirmed as a direct target gene of miR-122. STAT3 is a signaling mediator of the interleukin (IL)-6 and IL-10 family members and other cytokines, including leptin and granulocyte colony-stimulating factor. Previous studies have identified a negative effect of STAT3 in the type I IFN response (22,25). Ho and Ivashkiv (22) demonstrated that the overexpression of STAT3 in THP-1 cells downregulated IFN-α activated, STAT1-dependent genes, including interferon regulatory factor 1, CXC chemokine ligand (CXCL)9 and CXCL10. In addition STAT3 knockdown resulted in an increase in the expression of the same genes. Using gain-of-function and loss-of-function approaches, Wang *et al* (26) demonstrated that STAT3 negatively regulates the type I IFN-mediated response. Previous studies have also demonstrated that hepatic and circulating levels of miR-122 in HCV-infected patients were altered, indicating that HCV may regulate the host antiviral response by disturbing the expression of miR-122 (19,27,28).

The production of cell lines and transgenic organisms with continuous miRNA loss of function is enabled by a method termed the miRNA ‘sponge’. The sponge mRNA usually contains multiple target sites complementary to the miRNA of interest. To determine whether the HCV mRNA can act as an miR-122 sponge molecule, the HCV mRNA was overexpressed in Huh7 cells and the expression of miR-122 and STAT3 was detected. Although the expression of miR-122 was not significantly altered, the expression of STAT3 was rescued by HCV mRNA. These results indicated that HCV mRNA protected the miR-122 target genes by sponging miR-122.

In conclusion, the present study revealed another aspect of the association between HCV and miR-122. Previous studies have demonstrated that the expression of miR-122 enhances the propagation of HCV through genetic interaction with the 5′UTR of the HCV genome (29,30). miR-122 is a liver specific miRNA, the function of which is mainly associated with the maintenance of normal liver physiology. Therefore, it was hypothesized that miR-122 may be involved in the antiviral response of the liver. The present study initially predicted and confirmed that STAT3 is a target gene of miR-122. Subsequently, the wild type HCV genome RNA was found to protect the expression of STAT3 by absorbing miR-122, which functions as an RNA sponge. Subsequently, it was demonstrated that the HCV genome RNA inhibited the expression of IFN-α and IFN-β by upregulating the expression of STAT3, indicating that HCV RNA may act as an miR-122 sponge molecule that protects genes that are of benefit to the virus. The present study provided a further understanding of the complex roles of miR-122 in the interaction between HCV and host factors and supported the use of an miR-122 inhibition strategy in the treatment of HCV infection.
